# Lung cancer risk from radon exposure in dwellings in Sweden: how many cases can be prevented if radon levels are lowered?

**DOI:** 10.1007/s10552-015-0531-6

**Published:** 2015-02-13

**Authors:** Gösta Axelsson, Eva M. Andersson, Lars Barregard

**Affiliations:** Department of Occupational and Environmental Medicine, University of Gothenburg, Box 414, SE-405 30 Gothenburg, Sweden

**Keywords:** Radon, Lung cancer, Exposure, Prevention

## Abstract

**Purpose:**

Residential exposure to radon is considered to be the second cause of lung cancer after smoking. The purpose of this study was to estimate the number of lung cancer cases prevented from reducing radon exposure in Swedish dwellings.

**Methods:**

Measurements of indoor radon are available from national studies in 1990 and 2008 with 8992 and 1819 dwellings, considered representative of all Swedish dwellings. These data were used to estimate the distribution of radon in Swedish dwellings. Lung cancer risk was assumed to increase by 16 % per 100 becquerels per cubic meter (Bq/m^3^) indoor air radon. Estimates of future and saved cases of lung cancer were performed at both constant and changed lung cancer incidence rates over time.

**Results:**

The arithmetic mean concentration of radon was 113 Bq/m^3^ in 1990 and 90 Bq/m^3^ in 2008. Approximately 8 % of the population lived in houses with >200 Bq/m^3^. The estimated current number of lung cancer cases attributable to previous indoor radon exposure was 591 per year, and the number of future cases attributable to current exposure was 473. If radon levels above 100 Bq/m^3^ are lowered to 100 Bq/m^3^, 183 cases will be prevented. If levels >200 Bq/m^3^ are lowered to 140 Bq/m^3^ (mean in the present stratum 100–200 Bq/m^3^), 131 cases per year will be prevented.

**Conclusions:**

Although estimates are somewhat uncertain, 35–40 % of the radon attributed lung cancer cases can be prevented if radon levels >100 Bq/m^3^ are lowered to 100 Bq/m^3^.

## Background

The radioactive gas radon is a decay product of uranium normally found in rock and soils. It is the most important natural source of human exposure to ionizing radiation. The short-lived daughters of radon release ionizing radiation during radioactive decay and alpha particles emitted are capable of damaging DNA and increasing the risk of cancer. Studies of underground miners occupationally exposed to radon have demonstrated an increased risk of lung cancer among both smokers and nonsmokers [1–3], and the risk of exposure of miners has been found to be consistent with the risk of indoor exposure [4–6].

Radon can accumulate in buildings from ground leakage, which is the most common source of indoor radon. Radon-containing building materials, such as concrete and household water from deep-drilled wells, are other potential sources. Although the relationship between radon exposure in dwellings and lung cancer has occasionally been questioned [7], it is generally considered to be causal on an overall assessment of epidemiological studies, particularly in Europe and North America, and also in China [8–10]. Residential exposure to radon is considered to be the second most important cause of lung cancer after smoking [11].

Darby et al. [8] analyzed 13 European case–control studies and found a linear dose–response relationship, with no evidence of a threshold dose. The authors concluded that radon in homes accounts for about 9 % of all deaths from lung cancer in Europe.

Population exposure to radon can be lowered in existing residences by reducing the influx of radon from the ground or by removing radon-emitting building materials. Preventive measures can also be taken during the city-planning process when new residences are built. Preventive measures may be expensive, however, at least for existing buildings, and would require estimates of the number of lung cancer cases prevented. The single previous study we found that report such estimates was conducted in Ontario, Canada [12]. The aim of the present study was to estimate the number of lung cancer cases that would be prevented by reducing radon exposure in different scenarios in dwellings in Sweden.

Such an analysis requires data on the distribution of exposure in the population, the relationship between exposure and lung cancer risk, and time trends in the incidence rates.

## Methods

Measurements on indoor radon are available from two separate studies conducted in 1990 and 2008. The first was a large Swedish case–control study on lung cancer and residential radon levels [13]. It included 1360 cases and 2847 controls living in any of 109 Swedish municipalities selected for their assumed high or low radon levels in order to provide some contrast in residential radon levels. Radon was measured in all dwellings identified and available that had been occupied by study participants for at least 2 years since 1947 (a total of 8992 dwellings), covering almost all Swedish municipalities (259). For 5636 dwellings, the house type was recorded. The distribution was approximately log-normal with an overall geometric mean of 61 Bq/m^3^ and an arithmetic mean of 107 Bq/m^3^. We used data from those municipalities with at least 10 measurements (141 municipalities and 8217 dwellings), and for each municipality, the average radon exposure for each of the two house types (single-family house and apartment house) was estimated. For those municipalities that had less than 10 measurements, an estimated average exposure was used (82 Bq/m^3^ for apartments and 132 Bq/m^3^ for single-family houses).Thereafter, the national average exposure per person was estimated as a weighted average, with the weights equal to the number of persons living in single-family houses and apartments in each municipality. The distribution of radon exposure was estimated using these same weights. The arithmetic means were used in the calculations since the cancer risk in populations is associated with the population dose, which is represented by the arithmetic mean level multiplied with the size of the population.

The second study was conducted in 2008 by the National Board of Housing [14], using multiple-stage stratified sampling. Each of the municipalities in Sweden was classified into one of seven strata, and 30 municipalities were selected for measurements. From each of the 30 municipalities, a sample of dwellings was drawn, both single-family houses and apartments (in total 1819 dwellings). Each dwelling in which radon measurements were taken was assumed to represent dwellings of approximately the same age, temperature zone, and type of municipality (e.g., urban, rural). Weighting was used to calculate an average radon exposure per dwelling for seven strata with two house types in each (14 subgroups). The average exposure per person was estimated as a weighted average, with the weights equal to the number of people living in each of the 14 subgroups. The distribution of radon exposure was also estimated using these weights.

In the following analyses, the lung cancer risk was assumed to increase linearly by 16 % by increase in indoor radon by 100 Bq/m^3^, taking measurement error into consideration [8]. Estimates of future cases and of prevented cases of lung cancer use constant lung cancer incidence rates as well as changed rates over time, with the size of the population and age distribution assumed to be the same in different radon exposure classes and unchanged over time.

The number of lung cancer cases in relation to radon exposure is calculated in several steps. All estimated cases are divided into different intervals of radon concentration.

1. Estimate the radon concentration in dwellings around 1990.
2. Estimate the mean number of lung cancer cases per year in the period 2008–2012.
3. From (2), estimate the mean number of lung cancer cases per year in the period 2008–2012 had the radon concentration been 0 Bq/m^3^ in 1990.
4. Estimate the expected number of lung cancer cases in the future without radon exposure.
5. Estimate the radon concentrations in dwellings in 2010 based on measurements in 2008.
6. From (4) and (5), estimate the number of lung cancer cases in the future caused by the radon concentration in 2010.
7. Make assumptions about the magnitude of exposure after measures to reduce it.
8. Estimate the expected number of lung cancer cases in the future after this exposure reduction.
9. Estimate the prevented number of lung cancer cases from (8) minus (6).

## Results

The radon exposure in 1990 was estimated by multiplying the arithmetic mean in single-family homes and apartments in each municipality with the number of persons living in these types of dwelling in the municipalities. Measurements were not taken in 40 out of 284 municipalities, corresponding to 4.6 % of the total population in Sweden in 1990. For these municipalities, the weighted mean values from municipalities with at least 10 measurements were used. The estimated population-weighted radon exposure in 1990 was 113 Bq/m^3^ (arithmetic mean), and in 2008, it was 90 Bq/m^3^, where the exposure was 120 Bq/m^3^ in single-family homes and 55 Bq/m^3^ in apartments.

The distribution of radon exposure categories in the population in 1990 and 2010 is shown in Table 1. The data for 2010 are based on measurements made in 2008. Approximately 8 % of the population live in houses where the exposure is >200 Bq/m^3^.

**Table 1:** Estimate of population distribution in different categories of radon exposure in 1990 and 2010

Radon exposure (Bq/m^3^)	% in 1990	% in 2010
<50	27.6	53.8
50–100	26.8	21.2
100–200	38.0	16.5
100–150	34.7	9.8
150–200	3.3	6.7
200–400	4.9	5.5
>400	2.7	3.0

Table 2 shows the radon concentration in dwellings and the estimated number of lung cancer cases caused by radon in different exposure categories based on exposure data in 1990. The mean radon exposure per person is estimated as 113 Bq/m^3^ (line A, all), corresponding to a risk increase of 18 % (line B).

**Table 2:** Radon (Rn) concentration in dwellings and estimated number of lung cancer cases caused by radon in different exposure categories based on exposure data in 1990

Radon exposure Bq/m^3^	<50	50–100	100–150	150–200	200–400	>400	All
A. Mean Rn conc. in interval in 1990	29	75	130	173	275	747	113
B. Risk increase (%)	4.6	12.1	20.8	27.8	43.9	120	18
C. Distribution of population in 1990 (%)	27.6	26.8	34.7	3.3	4.9	2.7	100
D. Mean number of lung cancer cases in 2008–2012							3874
E. Expected mean number of cases in 2008–2012 if Rn conc. had been 0 in 1990	906	880	1139	108	161	89	3283
F. Expected mean number of cases in 2008–2012 at Rn conc. = 113 Bq/m^3^ in 1990	947	986	1376	138	231	196	3874
G. Calculated mean number of cases in 2008–2012 caused by Rn exposure in 1990	41	106	237	30	70	107	591

The mean radon exposure per person is estimated as 113 Bq/m^3^ (Line A, all). The increase in risk per 100 Bq/m^3^ is assumed to be 16 %, which corresponds to 18 % at 113 Bq/m^3^

A = data from Pershagen et al. [13], B = 16 % × A, C = data from Table 1, E = 3283 (3874/1.18) distributed according to C, F = E × B, G = F − E

During the period 2008–2012, the actual mean number of lung cancer cases per year was 3874 (line D). If the exposure in 1990 had been 0 Bq/m^3^, the expected mean number of cases in 2008–2012 would have been 3283 (line E, expected number of cases attributable to other causes). Thus, the estimated number of lung cancer cases attributable to indoor radon exposure in 1990 was 591 (line G), with the most cases in the radon interval 100–150 Bq/m^3^.

Table 3 shows the radon concentration in dwellings and the estimated number of lung cancer cases in the future caused by “current” radon exposure (based on exposure data from 2008). The current mean radon exposure per person is estimated as 90 Bq/m^3^ (Line A, all), corresponding to a risk increase of 14.4 %. Assuming that the baseline number of cases (cases attributable to other causes than radon) is again 3283 (line D), the estimated number of cases in the future, given the risk increase associated with the current exposure of 90 Bq/m^3^ (line B), is estimated as 3756 (line E).

**Table 3:** Radon concentration in dwellings and the calculated number of lung cancer cases in the future in different exposure categories based on exposure data from 2008 and the distribution of the population in 2010

Radon exposure Bq/m^3^	<50	50–100	100–150	150–200	200–400	>400	All
A. Mean Rn conc. in interval in 2008	29	69	122	166	277	725	90
B. Risk increase (%)	4.6	11.0	19.5	26.5	44.4	116	14.4
C. Distribution of population in 2010 (%)	53.8	21.2	9.8	6.7	5.5	3.0	100
D. Expected mean number of future cases if Rn conc. had been 0 in 1990 and 2010	1766	696	322	220	181	98	3283
E. Expected mean number of future cases at Rn conc. = 90 Bq/m^3^ in 2010	1847	773	385	278	261	212	3756
F. Calculated mean number of future cases caused by radon exposure in 2010	81	77	63	58	80	114	473

The mean radon exposure per person is estimated to 90 Bq/m^3^

A = data from National Board of Housing [14], B = 16 % × A, C = data from Table 1, D = 3283 (3874/1.18) distributed according to C, E = D × B, F = E − D

The expected number of cases in the future, based on the exposure data from 2008 (90 Bq/m^3^), is 3756. The calculations are made under the assumption of unchanged lung cancer incidence over time as well as unchanged population size in different age intervals and the same age distribution in all radon exposure categories.

The expected mean number of future cases if the exposure in 2008 had been 0 Bq/m^3^ was 3283, the same value as in Table 2. Thus, the estimated number of future lung cancer cases attributable to indoor radon was 473, with the largest number at >400 Bq/m^3^.

Table 4 shows the number of lung cancer cases that could be prevented if the mean radon concentration in the dwellings with the highest concentrations was lowered. In the first scenario, concentrations above 100 Bq/m^3^ are lowered to 100 Bq/m^3^. In this scenario, the future mean number of cases caused by radon would be reduced from 473 to 290. The number of prevented cases is thus 183, which is 39 % of the radon attributed lung cancer cases. In the second scenario, concentrations above 100 Bq/m^3^ are lowered to 69 Bq/m^3^, (the mean value in the interval 50–100 Bq/m^3^) and the mean number of cases caused by radon is 248, a reduction of 225 (48 %). In the third scenario, only concentrations >200 Bq/m^3^ are reduced to 140 Bq/m^3^ (the mean value in the interval 100–200 Bq/m^3^) and 131 future cases (28 %) would be prevented every year.

**Table 4:** Number of lung cancer cases that could be prevented if mean radon concentrations above 100 Bq/m^3^ were lowered to 100 or 69 Bq/m^3^, or if concentrations >200 Bq/m^3^ were lowered to 140 Bq/m^3^

Radon exposure Bq/m^3^	<50	50–100	100–150	150–200	200–400	>400	All
A. Distribution of population in 2010 (%)	53.8	21.2	9.8	6.7	5.5	3.0	100
B. Expected mean number of future cases if Rn conc. had been 0 in 1990 and 2010	1766	696	322	220	181	98	3283
C. Expected mean number of future cases at Rn conc. = 90 Bq/m^3^ in 2010	1847	773	385	278	261	212	3756
D. Calculated mean number of future cases caused by radon exposure in 2010	81	77	63	58	80	114	473
E. Calculated number of future cases, if Rn conc. above 100 Bq/m^3^ is lowered to 100 Bq/m^3^	1847	773	374	255	210	114	3573
F. Calculated mean number of future cases caused by radon after reduction in exposure to 100 Bq/m^3^	81	77	52	35	29	16	290
G. Calculated mean number of prevented cases after reduction in exposure to 100 Bq/m^3^	0	0	11	23	51	98	183
H. Calculated number of future cases, if Rn conc. above 100 Bq/m^3^ is lowered to 69 Bq/m^3^	1847	773	357	244	201	109	3531
I. Calculated mean number of future cases caused by radon after reduction in exposure to 69 Bq/m^3^	81	77	35	24	20	11	248
J. Calculated mean number of prevented cases after reduction in exposure to 69 Bq/m^3^	0	0	28	34	60	103	225
K. Calculated number of future cases, if Rn conc. above >200 Bq/m^3^ is lowered to 140 Bq/m^3^	1847	773	385	278	222	120	3625
L. Calculated mean number of future cases caused by radon after reduction in exposure >200–140 Bq/m^3^	81	77	63	58	41	22	342
M. Calculated mean number of prevented cases after reduction in exposure to 140 Bq/m^3^	0	0	0	0	39	92	131

A = data from Table 1, B = 3283 (3874/1.18) distributed according to A, C = B × risk increase (from B in Table 3), D = C − B, E = B × 1.16 in radon intervals >100 Bq/m^3^, F = E − B, G = D − F, H = B × 1.11 (1.16 × 0.69) in radon intervals >100 Bq/m^3^, I = H − B, J = D − I, K = B × 1.224 (1.16 × 1.4) in radon intervals >200 Bq/m^3^, L = K − B, M = D − L

## Discussion

The attributable fraction of lung cancer caused by residential radon exposure has been estimated in several studies. In addition to the estimation by Darby et al. [8] that 9 % of deaths in lung cancer in Europe is caused by radon exposure, such calculations have been reported from the USA [6], Canada [15], Germany [16], England [17], Romania [18], Spain [19], France [20], the Netherlands, and Sweden [21]. The estimated proportion of radon-caused lung cancer varied between 3 % in England and 20 % in Sweden.

One reason for the variation in attributable fraction in addition to different levels of exposure to radon may be differences in smoking habits. Available data in the literature suggest a strong interaction effect between radon exposure and smoking status, which mean that smokers are at a much higher risk of dying from radon-induced lung cancer than nonsmokers. The conclusion from the BEIR VI report [6] was that there is a sub-multiplicative interaction between radon and smoking and that any risk assessment for indoor radon needs to address the effect of radon on never smokers and ever smokers. Such risk assessments for radon exposure in relation to smoking status are seen in many studies [16, 20–27]. The attributable fraction of lung cancer caused by radon is estimated to be 3–4 times higher in nonsmokers than in smokers [16, 20]. Even so, the absolute risk in nonsmokers is small and, therefore, we have, in our calculation, assumed the same relative risk in smokers and nonsmokers.

The increase in risk per 100 Bq/m^3^ varies in different meta-analyses. Zhang et al. [10] found an increase in risk of 7 % in a meta-analysis of 22 studies comprising 13,380 cases and 21,102 controls. Darby et al. [8] reported in a meta-analysis of 13 European studies with 7148 cases and 14,208 controls an increase in risk of 16 % taking measurement error into consideration. We have chosen to use risk estimate from this study in the calculations of the number of radon-caused lung cancer cases in Sweden. Because the 95 % CI was quite wide (5–31), the true number of lung cancer cases caused by radon may thus differ substantially from the point estimate.

As in the study by Darby et al. [8], all age groups and all histological groups were included in our analysis. Darby’s study shows that the strongest association between radon exposure and lung cancer is found for small cell lung cancers, but increased risk of squamous cell carcinoma does not appear to be associated with radon. Squamous cell carcinoma accounted for 35 % of all lung cancers in Darby’s study, but only 18 % of cases in Sweden from 2008 to 2012. This suggests that the number of radon-caused lung cancer cases may be underestimated if Darby’s increased risk of 16 % per 100 Bq/m^3^ is used as the basis for calculations in Swedish data.

Estimates of the proportion of the population in 1990 and 2010 residing in a given radon interval are based on the radon epidemiological study of Pershagen et al. and measurements made by the National Board of Housing in 2008 [13, 14]. There is uncertainty in these estimates. For both 1990 and 2008, the standard error of the mean is 4 Bq/m^3^. In this study, 113 Bq/m^3^ is used as the population mean for residential radon in 1990. If we had used data from only the controls of the case–control study, the mean would have been 111 Bq/m^3^, since the exposure among the cases was approximately 5 % higher than that of the controls [8]. The bias is, however, negligible, since the estimated number of radon-caused lung cancers would be 584 (instead of 591) at a 16 % increased risk from a 100 Bq/m^3^ concentration.

A marked change in radon exposure happened in Sweden between 1990 and 2010. The proportion of residents living in the range of 100–200 Bq/m^3^ has more than halved from 38 to 17 %, and the percentage of residents with an exposure <50 Bq/m^3^ has doubled. However, the percentage of people living in houses >200 Bq/m^3^ is relatively constant. Possible reasons for the decreasing trend include decrease in the proportion of single-family homes, improved ventilation, radon remediation, and new construction techniques that reduce the risk of exposure. The change in exposure over this 20-year period can be assumed to reduce the number of radon-caused lung cancer cases from 591 to 473 cases per year. It should be noted that home energy efficiency interventions may have an opposite impact on future attempts to further reduce the indoor exposure to radon [28].

This study shows how many future lung cancer cases could be prevented by various alternative reductions in exposure. Because the mortality rate for lung cancer is close to the incidence rate, the number of lives saved from lung cancer approximates that of prevented cases. The population living in 8.5 % of the housing (>200 Bq/m^3^) represent 41 % of radon-caused lung cancer cases. If all dwellings above 200 Bq/m^3^ had their exposure lowered to 140 Bq/m^3^, 131 cases would be prevented each year and the proportion of radon-caused cases from exposure over 200 Bq/m^3^ would be reduced to 18 %.

If all exposures over 100 Bq/m^3^ were lowered to 100 Bq/m^3^, 183 cases would be prevented, and if all over 100 Bq/m^3^ were lowered to 69 Bq/m^3^, 225 cases would be prevented.

These calculations are based on several assumptions, one being that the lung cancer incidence rate is constant over time. A general decline in lung cancer incidence as a result of changes in smoking habits would mean that the number of prevented radon-related lung cancer cases will be lower than that the calculation shows.

Lung cancer in Sweden decreased slightly among men from around 45 cases per 100,000 in 1980 to about 40 in 2012. Among women, during the same period, the incidence rate increased sharply, from about 10 cases per 100,000 to about 40 (the same as men) in 2012. Since 2009, the incidence rate in women has begun to decline. The decrease in both men and women occurred first in the lower age groups; however, rates continue to increase in men aged over 74 and women aged over 64.

If current trends continue, the total lung cancer incidence rate will decrease by about 0.5 to 1 % per year. In 20 years, therefore, the total number of lung cancer cases may be 10–20 % lower than that today, mainly because of changed smoking habits, but also due to lower exposure to radon. The number of prevented radon-caused lung cancer cases may be about 15 % lower than that the calculations indicate. Due to the interaction effect between smoking and radon exposure, the impact of declining smoking is larger than remediation of high-radon houses on the radon-related lung cancer cases [29].

Other factors that affect the expected number of cases prevented in the future are population size and age distribution, i.e., when large birth cohorts reach the age when cancer is more common. In Sweden, in 2030 there will be 15,000–20,000 more 65-year-olds than there were 10 years earlier, while the number of 75-year-olds will be lower than a few years earlier. Thus, the number of lung cancers will depend on these population changes and how age-specific incidence rates change over time.

Chen (2013) [30] estimated the relationship between age, duration of exposure, and lifetime risk of lung cancer at different exposure levels using data from Canada. The increase in risk is relatively constant up to age 60 if the exposure starts during the first years of life. After 60 years of exposure, there is a flattening of the lifetime risk.

Estimates of the number of radon-caused lung cancers that can be prevented in different exposure classes are based on the assumption that smoking behavior is similar among the different exposure classes. Darby et al. [8] found in the analysis of 13 European case–control studies that the percentage of never smokers was highest in the exposure classes >400 Bq/m^3^. If this is also true in Sweden, the calculated number of radon-caused lung cancers in these groups is overestimated.

## Conclusion

Although there are some uncertainties in the estimates, approximately 25 to 30 % of lung cancer cases attributable to radon exposure could be prevented if all residential radon concentrations over 200 Bq/m^3^ were lowered to 140 Bq/m^3^; if all exposures above 100 Bq/m^3^ were lowered to 100 Bq/m^3^, about 35 to 40 % of these cases could be prevented.
